# Immunogenetics of Systemic Sclerosis

**DOI:** 10.3390/genes15050586

**Published:** 2024-05-05

**Authors:** Olga Gumkowska-Sroka, Kacper Kotyla, Przemysław Kotyla

**Affiliations:** Department of Rheumatology and Clinical Immunology, Medical University of Silesia, Voivodeship Hospital No. 5, 41-200 Sosnowiec, Poland; oag@poczta.onet.pl (O.G.-S.); kacper.kotyla@gmail.com (K.K.)

**Keywords:** systemic sclerosis, immune response, immunogenetics, single-nucleotide polymorphism, genome-wide association studies

## Abstract

Systemic sclerosis (SSc) is a rare autoimmune connective tissue disorder characterized by massive fibrosis, vascular damage, and immune imbalance. Advances in rheumatology and immunology over the past two decades have led to a redefinition of systemic sclerosis, shifting from its initial perception as primarily a “hyperfibrotic” state towards a recognition of systemic sclerosis as an immune-mediated disease. Consequently, the search for genetic markers has transitioned from focusing on fibrotic mechanisms to exploring immune regulatory pathways. Immunogenetics, an emerging field at the intersection of immunology, molecular biology, and genetics has provided valuable insights into inherited factors that influence immunity. Data from genetic studies conducted thus far indicate that alterations in genetic messages can significantly impact disease risk and progression. While certain genetic variations may confer protective effects, others may exacerbate disease susceptibility. This paper presents a comprehensive review of the most relevant genetic changes that influence both the risk and course of systemic sclerosis. Special emphasis is placed on factors regulating the immune response, recognizing their pivotal role in the pathogenesis of the disease.

## 1. Introduction

Systemic sclerosis (SSc) is a rare autoimmune connective tissue disease characterized by extensive fibrosis, vascular impairment, and immune system dysregulation [1]. Although skin fibrosis is the primary manifestation, vascular and internal organ damage contribute to the progressive nature and generally poor prognosis of the disease [2]. While previously considered solely as a “hyperfibrotic state” of the skin, recent advancements in rheumatology and clinical immunology have affirmed its autoimmune etiology, marked by abnormalities in immunocompetent cells and widespread autoantibody formation [3]. Similar to other connective tissue disorders, the precise pathophysiology of SSc remains subject to scientific debate. Presently, consensus suggests that disease development arises from a combination of environmental factors and genetic predispositions [4].

The prevalence of SSc varies geographically, ranging from 3.1 per 100,000 to 144.5 per 100,000 in specific ethnic populations, indicating non-random distribution. The disease predominantly affects middle-aged women, with a pooled prevalence of 33.9 per 100,000, compared to men, with a pooled prevalence of 6.0 per 100,000) [5]. Given the nearly universal presence of autoantibodies, SSc is unequivocally an autoimmune disorder, necessitating autoantibody testing as a critical component of patient evaluation. Anti-nuclear antibodies (ANAs) exhibit exceptionally high positivity rates, reaching 90–95% [6].

Detailed examination, employing the enzyme-linked immunosorbent assay (ELISA) method, enables the identification of scleroderma-specific antibodies targeting various antigens such as topoisomerase I (anti-TOPO/anti-Scl-70), kinetochore proteins (ACA), ribonuclear proteins (anti-U3 RNP/anti-fibrillarin, anti-U1 RNP, anti-U11/U12 RNP), RNA polymerase enzymes (anti-RNA polymerase III), and nucleolar antigens (anti-Th/To, anti-NOR 90, anti-Ku, antiRuvBL1/2, and anti-PM/Scl) [6].

Diverse subgroups of SSc exist, primarily classified on the basis of the area of the skin fibrosis. The classical classification, proposed by LeRoy et al. in 1988, distinguishes between limited cutaneous SSc (lcSSc), characterized by skin thickening not extending beyond the elbows and knees, and diffuse cutaneous SSc (dcSSc), where skin sclerosis affects proximal extremities and the trunk [7]. Additional subtypes include SSc without skin involvement (SSc sine scleroderma) and SSc as part of overlap syndrome, particularly with inflammatory myopathies [8]. Individuals with lcSSc may exhibit characteristic features such as calcinosis, Raynaud’s phenomenon, esophageal dysmotility, sclerodactyly, and telangiectasia, collectively termed CREST syndrome [9,10].

Most studies conducted to date have shown a link between the types of autoantibodies present and the subtype of the disease, as well as internal organ involvement [11,12]. In detail, using Hep-2 cells immunofluorescence enables the following three main fluorescence patterns to be distinguished in SSc patients:

1. Centromere pattern—(anti-centromere antibodies ACA) characteristics for lcSSc;
2. Speckled pattern suggesting the presence of anti-TOPO I (SCL-70) anti-RNA polymerase III. The presence of these antibodies suggests dcSSc;
3. Nucleolar pattern suggestive of anti-PM/SCl, anti-NOR90 U3RNP, anti-Th/To [6].

The seropositivity for SSc-specific antibodies helps to make a proper diagnosis. However, taking into account the fact that the role of ANAs in the pathophysiology of systemic sclerosis is the subject of controversy, the presence of ANAs cannot explain the pathophysiological background of the disease satisfactorily. Moreover, some scientists question the thesis of the pathogenic role of ANA in SSc, since others underline their presence in almost all patients and the fact that they are associated with disease activity and severity [13].

Studies have consistently shown an association between specific autoantibodies and SSc subtypes, as well as internal organ involvement. Environmental and occupational factors also significantly influence disease development, with various chemical compounds implicated, including silica, pesticides, organic solvents, and epoxy resins [14,15]. Additionally, bacterial and viral infections may contribute to disease initiation, albeit not as causative agents but rather as permissive factors. The role of infection may be explained by the direct interaction of the infectious agent with a population of immunocompetent cells, such as T and B cells, which leads to their functional instability and to the breaking of tolerance [16].

Several potential mechanisms have been proposed to date. Among them, molecular mimicry, and direct damage of tissues by viruses such as Parvovirus B19, cytomegalovirus (CMV), Epstein–Barr virus (EBV), retroviruses with subsequential release of pathogen-associated molecular patterns (PAMPs), and damage-associated molecular patterns (DAMPs) are often considered [17]. As a result, the immune system is tuned to an enhanced response. Moreover, the release PAMPs and DAMPS activate pattern recognition receptors (PRRs), mainly toll-like receptors (TLRs), which in turn facilitate the synthesis of interferon [18,19]. This finding may be of special importance, as interferon and interferon signature nowadays are recognized as important elements of SSc pathogenesis [20]. Immune effector mechanisms may then participate to cell damage and sustain inflammation until overt fibrosis occurs [21].

Inheritance is an important risk factor for the development of the disease [22]. This thesis is supported by the fact that the incidence of SSc is higher in first-degree relatives of persons suffering from SSc and strong family clustering, with an estimated risk of 1.6% versus 0.026%, is observed [23]. Limited information exists regarding the concordance rate for the disease in twins. While two studies indicated a relatively low concordance rate (4.7%), others demonstrated a significant frequency of concordance for the seropositivity for SSc specific autoantibodies and T cell activity, regardless of disease presentation. [24,25].

### Genomics of Systemic Sclerosis

As with other autoimmune diseases, the genetic context of the disease is mainly seen among mechanisms that potentially regulate immune responses. With many not fully understood mechanisms regulating the autoimmune response in systemic sclerosis, explanation of the genetic, or perhaps more precise, genomic, influence seems to be a proper way to understand the genetics risk of the disease as well as the genetic background of SSc.

## 2. Scientific Methods Used in SSc Studies

One of the most straightforward approaches to elucidating the influence of genetic material in a given subject is to compare the structure of information encoded in DNA between the subject and a control group. This method facilitates the detection of changes in single nucleotides, known as single-nucleotide polymorphisms (SNPs). However, a weak point of such studies is the absence of information regarding whether these SNPs may potentially lead to any clinical consequences. In other words, not all alterations at single positions in the DNA helix result in changes in the structure or function of the final protein, enzyme, molecule, etc. To address this limitation, alternative testing methods are currently being utilized. The candidate gene approach is a method that tests genes associated with a known disease or because they regulate important biological processes. This method simply allows for testing for particular SNPs with already known pathophysiological consequences.

The genome-wide association study (GWAS) is a method that scans for SNP across the entire genome of patients suffering from a given disease and compares them to healthy controls. The advantage of such an approach is to identify an SNP in the context of a given disease. A significant benefit of this methodology is the discovery of novel genes not previously suspected of being associated with diseases [26].

Finally, as recently tested, a novel genetic tool named the Genomic Risk Score has been successfully implemented to assess the risk for the development of a given disease. This tool was successfully tested in such diseases as SLE, sarcoidosis, vitiligo, the coronary heart diseases of schizophrenia, and systemic sclerosis [27,28,29].

Systemic sclerosis is not solely determined by genetics; however, considerable evidence suggests that genetic factors play a significant role in both disease initiation and progression. As systemic sclerosis exemplifies a prototypical autoimmune inflammatory disorder with a notable genetic influence, genes involved in the regulation of immune responses emerge as pivotal points of inquiry. These genes can be hierarchically categorized as follows:

- Major Histocompatibility Complex (MHC) Region HLA genes;
- Major Histocompatibility Complex (MHC) Region non-HLA genes;
- Non-Major Histocompatibility Complex (non-MHC) Region genes;
- Genes involved in cytokine synthesis regulation;
- Genes involved in immune signaling pathway regulation.

## 3. Major Histocompatibility Complex Region HLA Genes

Similar to other autoimmune inflammatory diseases, systemic sclerosis (SSc) exhibits a robust genetic association, particularly linked to a specific combination of MHC alleles. The locus responsible for encoding the major histocompatibility complex (MHC) molecule is situated on the short arm of chromosome 6 at band 6p21.3. This genomic region represents the largest cluster within the human genome, delineated into three primary sub-regions: class I, class II, and class III genes [30]. The MHC genes that are directly involved in the immune response fall into two classes, I and II, and encoded molecules which are structurally and functionally different. The class I genes code for the α polypeptide chain of the class I molecule; since the second component of MHC I molecule—the β chain of the class I molecule is encoded outside the HLA region by a gene on chromosome 15 [30,31]. The MHC system is the most polymorphic gene system known, and its gene products in humans are called HLA (human leukocyte antigens). There are 20 class I genes in the HLA region; however, taking into account the role in immune response and autoimmunity, only three of these, *HLA-A, B*, and *C*, the so-called classic, or class Ia genes, are recognized as main players in the immune response. Class I genes are expressed by most somatic cells, although the level of expression varies depending on the tissue [32].

In contrast to Class I, MHC II molecules are mainly expressed on the surfaces of immunocompetent cells as antigen-presenting cells, dendritic cells, B cells, activated T cells, macrophages, and thymic epithelial cells [30]. The main function of MHC molecules is to bind peptides (antigens) derived from pathogens and present then on the cell surface to be recognized by a specific clones of T cells. A fundamental function of the major histocompatibility complex (MHC) is to facilitate the recognition of antigens by the immune system. Antigens cannot be identified by the immune system in the absence of MHC molecules; therefore, they are only recognized within the context of MHC molecules. Numerous distinctive properties have been delineated to optimize the efficacy of the MHC recognition system [32]. There are multiple variants of each gene within the population as a whole, making it almost impossible for an invader to escape. The dark side of this system is, however, the high risk for mutation that may translate to autoimmunity-driven changes.

The accumulation of vitally important genes for immune response in the HLA region makes the HLA region the most important genetic cluster, encoding key molecules that orchestrate immune system functioning. Therefore, it is not surprising that a strong association between the HLA region and autoimmune disease (AID) has been proven for over fifty years [33].

Several GWAS performed in patients with systemic sclerosis confirmed that MHC is the strongest susceptibility loci for SSc. In line with this, twelve various gene loci, including *HLAB*, *C*, *DRA*, *DRB1*, *DRB5*, *DQA1*, *DQB1*, *DMB*, *DOA*, *DPA1*, *DPB1*, and *DPB2* have been shown to be linked with disease development. The most robust connections were identified in HLA-DRB1 *1104, HLA DQA1*0501, HLA-DQB1*0301, and *HLA-DQB1* [34]. In a comprehensive study conducted in 2010, which involved 1300 individuals diagnosed with SSc and 1000 healthy subjects encompassing diverse ethnic backgrounds such as Caucasian, African, and Hispanic Americans, it was revealed that the HLA class II alleles associated with the condition varied significantly across these ethnic groups [35]. Similar findings were observed in a Korean study of the HLA region (6p21.3), which indicated that *HLA-DPB1* and *DPB2* were strongly associated with SSc. Moreover, this association was most prominent in SSc patients with the anti-topo I antibody [36]. Several HLA studies have found a direct association between variations in MHC-encoding genes and the formation of disease-specific antibodies. Such an association was shown in HLA DPB1*1301 and HLA DRB1*1104 in Caucasian SSc patients with ATA autoantibodies [37]. In contrast, Reveille et al., in their multi-ethnic study encompassing Whites, Hispanics, and African Americans, demonstrated HLA-DRB1*08 as the prevalent HLA variant among African American SSc patients compared to local ethnic-matched control subjects. Furthermore, HLA-DQB1*0301 showed a significant association with SSc in both White and African American populations, as indicated by the Mantel–Haenszel summary chi-square analysis across all three ethnic groups [38].

An investigation into associations between *HLA-DRB1* and *HLA-DQB1* and specific autoantibody subsets revealed that only HLA-DRB1*11 exhibited an association with the anti-topoisomerase I (anti-topo I) autoantibody response across all three ethnic groups. Specifically, HLA-DRB1*1101 was more prevalent among White and African American individuals with anti-topo I autoantibodies, whereas a higher frequency of DRB1*1104 was observed in Hispanics compared to anti-topo I-negative systemic sclerosis (SSc) patients. Notably, within the Hispanic subgroup, an association between anti-topo I and HLA-DQB1*0402 alleles was identified [38]. At the moment it is not entirely clear whether the same subtypes of systemic sclerosis (diffuse vs. limited) may be linked to the specific HLA variants. At the moment, such indirect data were obtained from studies where the frequencies of ATA vs. ACA autoantibodies were assessed. Keeping in mind that ATAs are generally linked to the diffuse form of the disease and that ACAs are linked to the limited form, HLA-DRB1*01, HLA-DRB1*04, HLA-DRB1*08, HLA-DQB1*0501, HLA-DDQB1*26 haplotypes linked to the presence of ACA antibodies may suggest an association with limited systemic sclerosis [35,37,38].

A recent study from Spain shed new light on HLA gene frequencies in two main subtypes of SSc. For lcSSc, six leading classical alleles were identified as being independently associated. Moreover, among them, HLA-DQA1*02:01 has been shown to be exclusively associated with lcSSc. Similar findings were observed in studies on dcSSc, where four classical alleles were linked with this subphenotype when compared with healthy individuals, with HLA-DQA1*05:01 exclusively associated with dcSSc [35]. Additionally, a combination of HLA alleles may at least partially influence the antibody profile. In line with this, HLA-DRB1*0404, HLA-DRB1*11 HLA-DQB1*03 characterized patients with SSc and anti-RNA polymerase III seropositivity [35]. Quite recently, a study from Thailand revealed higher frequencies (AFs) of HLA-DRB1*15:02:01, DRB5*01:02:01, DQB1*05:01:24, DPB1*13:01:01, and DQA1*01:01:01 in all SSc patients as well as in SSc subjects with positive ATA, but with negative ACA (SSc/ATA+/ACA-) [39]. The authors also identified DPB1*13:01:01 as the most susceptible allele [39]. Notably, not all HLA genes confer a risk for the development of systemic sclerosis. In a study conducted in Japan, Furukawa et al. demonstrated that certain HLA alleles, namely DRB1*13:02, DRB1*14:06, DQB1*03:01, and DPB1*02:01, were independently associated with protection against susceptibility to SSc [40]. A similar conclusion was drawn in an Australian–American independent and meta-analyzed cohort of 1,465 SSc patients. According to the results of the study, there was a strong disease-risk association of HLA DPB1*13:01 in the diffuse type of SSc, whereas a protective effect was shown with regard to HLA-DRB1*07:01, HLA-DQA1*02:01, HLA-DQB1*02:02, HLA-DRB4*01:01 HLA-B*44:03, and HLA-C*16:01. Moreover, in the study, protective alleles were associated with limited SSc [41].

The association between MHC class I and systemic sclerosis is considerably less well-elucidated. Recent research suggests that certain HLA class I alleles can function as ligands for killer Ig-like receptors (KIR), thereby modulating the activity of natural killer (NK) cells [42]. The protective HLA-B*44:03 allele against limited cutaneous systemic sclerosis is a member of the *HLA-Bw4 KIR* ligands and interacts with the specific receptor KIR3DL1. Recent findings indicate that fever-inducing *KIR3DL1+* SSc individuals express the HLA-Bw4 inhibitory ligand compared to *KIR3DL1*+ controls [41]. This discovery could elucidate the direct interplay between the composite of HLA class I and the function of NK cells, which directly correlates with an increased risk of SSc development, although some studies in the literature contradict this assertion [43,44].

## 4. Non-HLA Complex Genes

Several lines of evidence suggest that innate immune system in SSc become one of the main players responsible for the onset and progression of the pathophysiological process in the disease [18,19].

The innate immune system is widely understood as the initial line of defense against pathogens and chemical or mechanical challenges to the body. However, it lacks the ability to distinguish between foreign (xeno) and self-antigens, potentially triggering autoimmune responses [45,46,47]. It is not surprising that genes directly involved in innate responses are able to modulate the disease course, being at least partially responsible for the onset of the disease.

## 5. The Interferon Regulatory Factor Genes

Numerous studies have validated the involvement of interferons in the pathogenesis of SSc [21,48,49]. Furthermore, a substantial body of evidence has established interferons as highly pleiotropic cytokines that play a pathogenetic role in diseases such as systemic lupus erythematosus, rheumatoid arthritis, idiopathic inflammatory myositis, and systemic sclerosis [50,51,52,53,54,55]. The activity of interferons is precisely regulated by interferon regulatory factors (IRFs). The mammalian family of IRF genes consists of nine IRFs, namely *IRF1, IRF2, IRF3, IRF4/PIP/LSIRF/ICSAT, IRF5, IRF6, IRF7, IRF8/ICSBP,* and *IRF9/ISGF3γ* [56]. All IRF proteins have a conserved amino-terminal DNA-binding domain (DBD), which recognizes a DNA sequence element named the interferon sensitive response element (ISRE) in the gene promoters of type I IFN-, type III IFN-, and IFN-stimulated genes (ISG), and thus regulates the expression of interferon-related genes [56,57,58].

Considering the fundamental role of interferon regulatory factors (IRFs) in modulating interferon-based immune responses, recent evidence has increasingly implicated several IRF genes in the pathogenesis of systemic sclerosis. Historically, IRF5 was the first gene identified through candidate gene studies across diverse ethnic populations to potentially contribute to SSc pathogenesis. Single-nucleotide polymorphisms (SNPs) located in the IRF5-TNPO3 region exhibited the strongest associations beyond the human leukocyte antigen (HLA) region, a finding corroborated by numerous SSc Genome-Wide Association (GWA) and Immunochip studies [59]. Interestingly, SNPs identified through candidate gene studies, including rs2004640, rs2280714, rs10488631, and rs10954213, were found to be associated with an increased risk of SSc development. Moreover, recent research has linked the rs2004640 polymorphism with the occurrence of interstitial lung diseases in SSc patients [60]. More recently, the rs2004640T allele and rs10488631 C allele of IRF5 have been identified as risk factors for SSc. Regarding the implications of these findings, associations have been noted between IRF5 rs10488631 and declines in forced vital capacity (FVC) and diffusing capacity of the lungs for carbon monoxide (DLCO) over time, although neither remained significant after Bonferroni correction [61]. The involvement of other IRFs in SSc pathogenesis remains less understood. Currently, intensive investigation is focused on *IRF1, IRF4, IRF7,* and *IRF8* regarding their potential roles in SSc development. To date, only one investigation has explored the involvement of IRF1 in SSc pathogenesis. In 2020, Gonzales-Serna et al. conducted a cross-disease meta-analysis incorporating GWAS data from 5734 SSc patients, 4588 Crohn’s disease (CD) patients, and 14,568 controls of European descent. They identified several shared loci implicated in the development of both systemic sclerosis and Crohn’s disease. Notably, the SNP within the *IRF1* locus (rs2548998) was found to play a role in both SSc and CD [62].

The role of IRF4 in SSc seems to be a little controversial, as the first GAWS failed to confirm a link between SNP in EXOC2- IRF4 to SSC [63]. Finally, this link has been confirmed indirectly in the other study by identification of IFR4 as a common susceptibility gene shared between rheumatoid arthritis and SSc [64].

The role of the *IRF8* gene in the pathogenesis of SSc was proposed in 2011. Two main SNPs, namely rs11642873, which was associated with limited (centromere-positive) SSc in Caucasians in Europe and North America [65], and rs 2280381, in European and Japanese populations, were discovered [66]. Since then, several SNPs linked to SSc have been identified. At the current level of knowledge, these include: rs11117432, rs11644034, rs12711490, rs7202472, and rs11117420 [34]. The role of *IRF8* in profibrotic activity has been recently suggested by Ototake et al. [67]. *IRF 8* is the main antifibrotic regulator and its level is downregulated in the PBMCs of patients with systemic sclerosis and is negatively correlated with modified the Rodnan total skin thickness score [67]. Thus, findings in genetic studies may explain profibrotic activity in SSc.

## 6. STAT Genes and JAK-STAT Pathway

The discovery of the non-receptor tyrosine kinase family (Janus kinases-JAK), a group of intracellular enzymes essential for the proper functioning and activation of class I and class II receptor families translated to the discovery of Janus kinase inhibitors, a group of drugs that have demonstrated their safety and efficacy in several diseases with an autoimmune background. Apart from their well-known efficacy in rheumatoid arthritis [68], their position in the treatment of several autoimmune diseases, including systemic sclerosis, has been intensively tested [69,70].

In the field of human biology, it has been observed that the JAK family consists of four distinct members, namely, JAK1, JAK2, JAK3, and TYK2. Each of these members has been found to be specifically associated with different types of cytokine receptors. When a cytokine (ligand) binds to its corresponding receptor, it initiates receptor activation, although for the complete activation process, the formation of a complex involving JAK kinase is necessary. This active complex, consisting of the ligand, receptor, and JAK kinase, plays a crucial role in facilitating the binding of other transmission molecules, specifically known as STATs (signal transducer and activator of transcription). The signal transducer and activator of transcription (STAT) genes are a group of transcription factors activated by JAK kinases. At the current level of knowledge, seven STATs have been identified: STAT1, STAT2, STAT3, STAT4, STAT5a, STAT5b, and STAT6 [71]. Stat molecules are responsible for the signaling of several key pro- and anti-inflammatory cytokines. In detail, STAT1 homodimers signal type II interferon activity, since heterodimer STAT1-STAT2 is responsible for the transmission of IFN type I signaling.

Another player in this field is STAT 4. STAT4 activity seems to be essential for driving the inflammatory response, since different combinations of STAT4 are activated by a variety of cytokines, including interleukin (IL)12. The role of STAT4 polymorphisms in the development of SSc was first suggested by several candidate genes studies and then confirmed by GWAS immunochip and meta-analyses [59]. These studies addressed the role of SNP in the development of the disease. In line with this, the SNP of *STAT4* intron 3 (rs7574865) has been found to be associated with the limited cutaneous form of systemic sclerosis [72]. Followed by this observation, made by Ruead et al., several confirmation studies have been conducted. The initial observation made by the Spanish team has also been extended to the diffuse form of the disease, the presence of interstitial lung disease (ILD), cardiac injury (CI), and seropositivity for anti-topoisomerase I antibodies (ATA) [73]. Additional data have emerged from the French study conducted by Diede et al. that further supports previous findings. Within a cohort of 1855 French Caucasian individuals, the authors observed a strong association between the *STAT4* rs7574865 variant and systemic sclerosis (SSc). Notably, this association was not limited to a specific SSc phenotype, implying the broader involvement of the STAT4 gene in the overall susceptibility to the disease [74]. Subsequent to these results, the role of the SNP rs7574865 has been examined in a Japanese population, which entirely corroborated the previous findings from European studies regarding its role in systemic sclerosis (SSc) development. However, in contrast to the results observed in Caucasians, a significant association between rs7574865 and the formation of anticentromere antibodies (ACA) was demonstrated [75].

Extensive investigations into STAT4 polymorphisms have led to the identification of additional single-nucleotide polymorphisms (SNPs) within the STAT gene family. Specifically, apart from the previously reported SNP in rs7574865 gene, four additional SNPs—namely, rs11889341 (A), rs8179673 (C), rs10181656 (C), and rs6752770 (C)—were discovered in 2009 [76].

Less is known about the role of *STAT4* gene polymorphisms in the development of SSc-related complications. Among these, interstitial lung disease, the main killer in SSc, plays a role. As it was suggested recently, the STAT4 rs7574865 allele may play a protective role against the development of interstitial lung disease [61].

Another player in this field is STAT3. Considering its wide-ranging regulatory influence, it is understandable that the dysregulation of STAT3 signaling has been implicated in the development of numerous diseases, including systemic sclerosis [77]. STAT3 undergoes activation in response to various pro-inflammatory cytokines, including interleukin-6 (IL-6) but also by strong profibrotic factors such as TGF [77,78]. In SSc patients, the expression of the *STAT3* gene was observed to be reduced in comparison to individuals without SSc; however, differences were statistically nonsignificant. On the other hand, STAT 3 is deeply involved in the signaling from ana-proinflammatory cytokines, such as IL6 IL21, IL22 and IL23, and indirectly regulates the fate of Th17 cells, which are directly responsible for SSc-related fibrosis. The role of STAT3 in this context remains less understood. A single study conducted in Spain has shed light on the potential involvement of the STAT3 gene polymorphism, specifically the SNP STAT3 (rs4796791) locus, in the development of systemic sclerosis (SSc) [62]. However, further research is needed to fully elucidate the impact and significance of STAT3 in the pathogenesis of SSc [79].

In contrast to other hematological and autoimmune disorders, information regarding the involvement of JAK kinases in systemic sclerosis is scarce. Within the JAK family, TYK2 stands out as the sole member facilitating the signaling of IL-12 family cytokines, including IL-12 and IL-23, and is recognized as a shared genetic risk factor for various autoimmune conditions. To date, only four single-nucleotide polymorphisms (SNPs) in the TYK2 gene (rs34536443, rs35018800, rs12720356, rs2304256) have been identified as being associated with systemic sclerosis [79].

## 7. Toll-like Receptors

Recent studies on the pathogenesis of systemic sclerosis underline the role of Toll-like receptors (TLR) as the first line of pathogen/autoantigen recognition in the development of the disease [80]. However, the precise role of TLR is still controversial. In line with this, the role of TLRs polymorphism is not fully understood. To investigate the role of TLRs polymorphism in a discovery cohort comprising 452 SSc patients, Broen et al. genotyped 14 polymorphisms in the genes for *TLRs 2, 4, 7, 8,* and *9*. They observed that a rare functional polymorphism in TLR2 (Pro631His) was associated with antitopoisomerase (Scl-70) positivity as well as with the diffuse subtype of the disease. Moreover, the functional analysis revealed that monocyte-derived dendritic Pro63His variant cells are characterized by high inflammatory potential and synthesized increased levels of inflammatory mediators (TNFα and IL6) [81].

## 8. The Nuclear Factor κB

The nuclear factor κB (NF-κB) pathway regulates many aspects of innate and adaptive immune responses, and its main role is to regulate the expression of many proinflammatory genes responsible for cytokines and chemokines synthesis. As the bridge of innate and adaptive immune response, NF-κB is deeply involved in the regulation of activation differentiation and survival of T cells as well as in the regulation of inflammasome activity [82,83,84]. Dysfunction of the NF-κB pathway can be observed in both the early and late stages of SSc [85]. Polymorphisms in genes associated with the NF-κB pathway have been implicated in systemic sclerosis susceptibility. Specifically, SNPs located within the promoter region of the *NFKB1* gene, namely rs230534, rs4648133, and rs1599961, have demonstrated associations with systemic sclerosis in European, Turkish/Iranian, and Chinese Han populations [86,87,88]. Another gene polymorphism identified in the NF-κB pathway among systemic sclerosis patients is within the TNFα-induced protein 3 gene (*TNFAIP3*), which encodes TNFα-induced protein 3, also known as A20 protein. A20 protein serves as a negative regulator of the TNF-induced NF-κB pathway. Three intronic variants and one exonic variant of the *TNFAIP3* gene have been linked to systemic sclerosis (rs5029939, rs58905141, rs117480515, rs2230926) [89]. Moreover, rs5029939 has been associated with the most severe form of the disease. Fibroblasts carrying the *TNFAIP3* rs58905141 variant exhibit heightened expression of matrix metalloproteinases MMP1 and MMP3, contributing to increased fibrosis [90]. Moreover, the A20 protein regulates TGFβ-induced fibroblasts transformation and collagen synthesis in fibroblasts cultures, acting as a negative regulatory factor [91]. The other regulatory factor in this field is the TNFAIP3-interacting protein 1 (TNIP1) that regulates TNFAIP3 activity. TNIP1 acts as a negative modulator of inflammatory response; its loss or dysfunction promotes the expression of numerous proinflammatory cytokine and chemokine genes [92]. In line with this, the TNIP1 gene has been found to be associated with SSc [86,93].

## 9. Macrophage Migration Inhibitory Factor (MIF)

Despite being initially identified as the protein secreted by activated T lymphocytes, the macrophage migration inhibitory factor (MIF) is indeed produced by a wide range of immune cells, including macrophages, endothelial cells, and fibroblasts. The biological function of the cytokine goes far beyond the regulation of macrophage motion and includes the control of cell proliferation, activation of T lymphocytes, and induction of the synthesis of proinflammatory cytokines, including TNFα, IL1, IL6, and IL8 in immunocompetent cells [94]. The gene encoding MIF resides on chromosome 22 (22q11.23) within the human genome and comprises three exons. (of 107, 172, and 66 bp, respectively) and two introns (188 and 94 bp). This gene encodes a low molecular-weight protein composed from 114 amino acid residues. MIF can interact with several membrane receptors such as cluster of differentiation 74 (CD74), chemokine (CXC motif) receptor CXCR2, CXCR4, and CXCR7 [94]. More than two decades ago, the role of MIF in SSc was shown [95]. Currently, some studies have linked MIF concentration to particular SSc presentations, such as pulmonary arterial hypertension (PAH), impaired lung function, and ACE inhibitor usage, although not all studies have confirmed this [96]. Three genetic studies have suggested that MIF polymorphisms are linked to SSc disease severity in SSc in Whites from Europe and North America [97,98,99]. In particular, the association of the MIF promoter variant rs755622*C allele with systemic sclerosis (SSc) has been demonstrated in a substantial cohort of SSc patients of European descent [98,99]. Moreover, variant rs755622*C was associated with the higher risk of developing PAH in subjects suffering from dcSSc [98].

## 10. The Interleukin-1 Receptor Associated Kinases

The IRAK family comprises four members: IRAK-1, IRAK-2, and IRAK-4, which are expressed in various human immune cell types, and IRAK-M, predominantly found in monocytes and macrophages [100].The role of IRAK is to transmit signals from IL1 receptor and TLRs and then to regulate NF κB, MAPK, STAT, and IRF pathways [101]. IRAK1 is a protein of 712 amino acids and has a molecular mass of about 85kDa. IRAK1 is encoded by the *IRAK1* gene located at the X chromosome (Xq28). It is supposed that the location of the *IRAK1* gene on the X chromosome may at least partially explain the higher risk of some autoimmune diseases among females [102]. To date, the role of sex chromosomes in SSc susceptibility is still a matter of controversy. Yet, according to findings from a comprehensive European study, an *IRAK1* haplotype containing the functional variant 196Phe (rs1059702), situated on Xq28, was identified as conferring susceptibility to systemic sclerosis (SSc). Moreover, the study confirmed a strong association between *IRAK1* haplotype and dcSSc, anti–topo I–positivity, and SSc-related interstitial lung disease [103].

## 11. Adaptive Immune System

The adaptive immune response acts as the secondary defense line for the host’s defense against invaders. This crucial task is primarily carried out by lymphocytes, which possess the ability to recognize the molecular structures of pathogens and initiate an effective immune response [104]. However, for this system to function optimally, it is equipped with a diverse array of receptors capable of recognizing antigens. Among these receptors, one of the key players is the T cell receptor (TCR), which is associated with the CD3 molecule, functioning as a co-receptor on T cells [105]. Ensuring the proper functioning of this complex is the CD3ζ protein (also known as CD247), a transmembrane signaling adaptor protein. CD3ζ facilitates various downstream signaling cascades, ultimately leading to the full activation of T cells [106,107].

**Figure 1:**
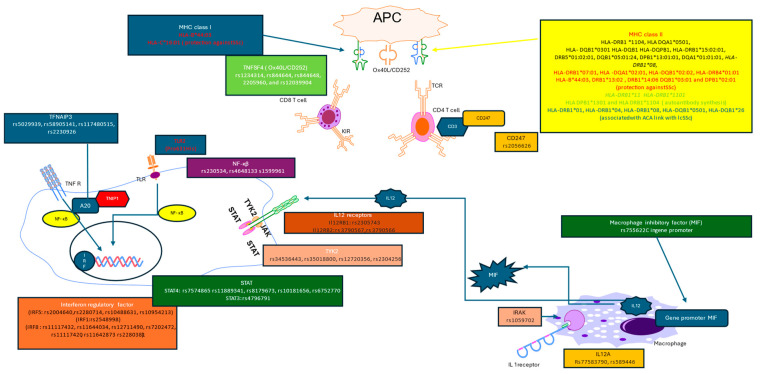
Systemic sclerosis, recognized as a prototypical autoimmune disease, is intricately governed by a myriad of autoimmune mechanisms. Among these, the major histocompatibility complex (MHC) class I and class II play a pivotal role in the precise presentation of both antigens and autoantigens. Various combinations of MHC I and MHC II alleles have been correlated with the susceptibility to disease onset and the manifestation of specific disease subtypes. Antigen presentation occurs within the context of MHC class I and II molecules, subsequently eliciting responses from CD8 and CD4 T cells, respectively. Co-receptor molecules, such as Ox40L for CD8 T cells, and CD2 and CD247 for CD4 cells, orchestrate proper co-stimulation, culminating in the orchestration of the final immune response. Genetic variability within the interferon system has been extensively documented. Commencing from genes responsible for Toll-like receptor (TLR) encoding, traversing through the intricate signaling cascades mediated by NFκB, and culminating in genetic variations in interferon regulatory factors. A multitude of cytokines are implicated in the pathogenesis of systemic sclerosis, including those operating via the JAK/STAT signaling pathway (e.g., IL-12). Genetic polymorphisms within genes encoding IL-12 receptor, TYK2, and STAT molecules have been associated with the susceptibility to systemic sclerosis development. Similar associations are observed concerning other cytokines such as IL1 (IRAK SNP) and the macrophage migration inhibitory factor (gene promoter of MIF).

The *CD247* gene is situated on the human chromosome 1q24.2. SNPs within the *CD247* gene, encoding the CD247 protein, have been implicated in many connective tissue disorders, including systemic sclerosis (SSc) [108,109,110,111]. Notably, the involvement of the *CD247* rs2056626 SNP has been observed in various studies, although not all investigations have corroborated this finding [63,111,112,113,114]. Recent propositions have suggested that certain genes linked to SSc, such as *IRF8, STAT4*, and *CD247*, exhibit interactions that are specific to particular cell types. Particularly, significant interactions between SNPs and the *CD247* promoter have been exclusively observed in T helper cells. Additionally, heightened expression of the *CD247* gene has been documented in CD4+ T cells in comparison to CD14+ monocytes [115] (Figure 1).

## 12. IL12 Axis and Its Receptors

The cytokine network observed in systemic sclerosis (SSc) exhibits a strong dependence on both the type and stage of the disease [100]. Early stages are typically characterized by heightened inflammation, marked by a predominant Th1 response [116]. Conversely, in later stages of the disease, there is a shift towards a Th2 response [116,117]. In line with this, interleukin-12 (IL-12), recognized as one of the most potent proinflammatory cytokines in SSc, appears to primarily influence the early stages (inflammation) of the disease. Moreover, elevated levels of IL-12 have been detected in SSc patients [118], implicating its role in the pathophysiology of the condition. Conversely, an increase in serum IL-12 concentrations has been associated with the attenuation of fibrotic processes in advanced stages of the disease [119].

The Immunochip study, conducted by Mayes in 2014, facilitated the identification of three non-HLA loci associated with systemic sclerosis (SSc) [120]. Among these loci, the authors demonstrated the association of a SNP rs77583790, located in the intergenic region between *SCHIP1* and *IL12A* at 3q25, with SSc [121]. Furthermore, the role of IL12 polymorphisms has been supported by genome-wide association studies (GWAS), leading to the discovery of a novel non-coding SNP of *IL12A*, rs589446 [86].

However, the precise mechanism by which IL12A may contribute to the pathogenesis of SSc remains controversial. A recent study examining long-range interactions between SSc-associated GWAS SNPs and the promoter of structural maintenance of chromosome 4 (*SMC4*) in CD14+ monocytes—a promoter directly implicated in inflammatory innate immune responses—failed to observe interactions between SSc-associated GWAS SNPs and the promoter of this gene [115].

The significance of the IL-12 axis in systemic sclerosis goes beyond the gene encoding IL-12, with considerable attention directed towards IL-12 receptors. Notably, IL12RB1 rs2305743 and SNPs in *IL12RB2* (rs3790567 and rs3790566) have been associated with SSc [121,122,123]. Moreover, the role of *IL12RB2* is not exclusive to SSc, as evidenced by the involvement of SNP rs6659932 in both SSc and Crohn’s disease [62]. This suggests a pleiotropic role of IL-12 and its genetic mutations in the development of several autoimmune diseases [116].

## 13. TNF Superfamily Member 4 (*TNFSF4*)

Co-stimulation represents a critical aspect of the immune response, yet dysregulation in this process may serve as an initial trigger for the development of autoimmunity. Co-stimulation involves the modification of signals provided to T cells upon recognition of their specific antigen, mediated by signals from membrane-bound molecules [124]. This intricate process necessitates the interaction of numerous molecules with their corresponding receptors on immune cells. Notably, OX40 ligand (OX40L or CD252), expressed on antigen-presenting cells, has garnered significant scientific interest due to its involvement in the regulation of T cell division, survival, and function [125].

OX40L is encoded by the *TNF superfamily member 4* (*TNFSF4*) gene, and polymorphisms within this gene have been linked to various autoimmune diseases, including Sjögren’s syndrome, systemic lupus erythematosus, and systemic sclerosis (SSc). Several single-nucleotide polymorphisms (SNPs), particularly within intronic and regulatory regions of the *TNFSF4* gene (e.g., rs1234314, rs844644, rs844648, 2205960, and rs12039904), have been associated with SSc in genome-wide association studies (GWAS) and replicated in candidate gene approaches within European SSc populations [126,127]. Consequently, it has been postulated that *TNFSF4* may serve as a susceptibility gene for SSc [128].

Furthermore, recent findings indicate that the *TNFA C* rs1799724 variant is associated with an elevated risk of SSc. Additionally, based on the detection of cancers within their cohort, the authors have speculated that the CT rs1799964 and AG rs361525 genotypes may exacerbate cancer susceptibility in individuals with SSc [129].

## 14. The Role and Polymorphism of Interleukin-6 (IL6)

Interleukin-6 (IL-6), recognized as one of the most potent proinflammatory cytokines, assumes a distinctive role in the progression of systemic sclerosis, contributing significantly to both vascular damage and fibrosis development [130]. In the initial phases of SSc pathogenesis, IL-6 exerts influence by activating vascular endothelial cells and inducing apoptosis [131,132]. Additionally, it triggers the liberation of damage-associated molecular patterns (DAMPs), perpetuating the inflammatory response and autoimmunity [133]. Furthermore, IL-6 plays a pivotal role in fostering fibrotic alterations, directly facilitating the differentiation of fibroblasts into myofibroblasts [134]. The polymorphism of the IL-6 gene has been investigated in various clinical trials, with particular attention directed towards the rs1800795 (-174C/G) variant, which is frequently associated with elevated production of this cytokine [135].

Notably, clinical findings from a study involving 102 individuals with systemic sclerosis (SSc) demonstrated heightened IL-6 gene expression in carriers of the G allele. Of particular interest is the observation that this genetic polymorphism of IL-6 was specifically correlated with the presence of gastrointestinal manifestations, while no significant associations were found with skin and lung damage [136]. Other data obtained after studying 20 cases with SS support the link between IL-6 polymorphism, especially the homozygous *GG* form, which was linked with the most severe form of the disease [137].

The association of the *IL-6* rs1800795 polymorphism with disease severity was further confirmed in the investigation conducted by Beretta et al., who identified this polymorphism as a potential risk factor for the development of limited cutaneous systemic sclerosis (lcSSc) [138].

The involvement of *IL-6* polymorphism in the pathogenesis of diffuse cutaneous systemic sclerosis (dSSc) is subject to more controversy. According to findings from the same study, which employed a non-parametric computational algorithm known as multifactor dimensionality reduction, the role of the *IL-6* ANT565G polymorphism is part of a complex interplay with other single-nucleotide polymorphisms, specifically, *IL1R* Cpst1970T and *IL-10* C-819T [138]. Thus, it can be considered a component of the broader genetic milieu contributing to the development of the disease.

## 15. Conclusions

Immunogenetics, a branch of genetics concerned with the impact of genetic alterations on immune system functionality and their implication in the development of autoimmune disorders, is currently a subject of considerable scientific interest. Hence, it is unsurprising that systemic sclerosis, a prototypical autoimmune condition, is notably influenced by genetic modifications affecting the structure and operation of the immune system. Genetic variability can influence the nature and trajectory of the disease in various ways, commencing with polymorphisms in both HLA and non-HLA genes, which significantly alter the mechanism of autoantigen presentation and the recognition of DAMPs particles by immunocompetent cells, ultimately culminating in the production of a spectrum of cytokines directly implicated in the initiation and perpetuation of the disease process.

We are currently accumulating data to potentially elucidate the role of genetic variations in the development of specific subtypes of systemic sclerosis (dcSSc vs. lcSSc), while also attempting to establish links between genetic alterations and the onset of SSc-related complications such as lung fibrosis, pulmonary arterial hypertension, or gastrointestinal involvement. Presently, advancements in genetics enable us to assess the risk of developing a particular type of the disease and speculate on the risk of disease-related complications. Unfortunately, we are currently only scratching the surface, as we are testing only a limited subset of factors potentially implicated in the pathomechanism of the disease. With advancements in whole genome studies, we anticipate being able to explore genes that are currently beyond the scope of our investigations. It seems reasonable to expect that this approach will provide new insights into the role of genetic variation in the pathogenesis of immune-mediated diseases, including systemic sclerosis.
